# A Giant Epidermal Cyst in the Gluteal Region: A Case Report

**DOI:** 10.7759/cureus.34266

**Published:** 2023-01-27

**Authors:** Rahul Rajendran, Sajika P Dighe

**Affiliations:** 1 Department of General Surgery, Jawaharlal Nehru Medical College, Datta Meghe Institute of Higher Education and Research, Wardha, IND

**Keywords:** mri- magnetic resonance imaging, ultrasonography (usg), gluteal region, giant, epidermal inclusion cyst

## Abstract

Epidermal cysts are the most commonly occurring cysts in the subcutaneous plane and are usually asymptomatic, small, and slow growing. When the size of an epidermal cyst is greater than 5 cm, it is considered a giant epidermal cyst. Common etiologies include sun-damaged skin and acne vulgaris, and they can appear anywhere in the body, but preferably over the face, neck, and trunk. Unusual sites include the breast, penis, spleen, bones, subungual region, palms, soles, and buttocks. In this report, we presented the case of a 31-year-old female having a large, painless, gradually progressing swelling for two years in the left gluteal region, which was insidious in onset and slow-growing. The patient eventually described a discomfort that made it impossible for her to sit for long hours or sleep in a supine position. Clinical examination revealed circumscribed mass over the left gluteal region from which diagnosis of giant lipoma was confirmed, but due to its large size and involvement of the whole left buttock, we considered it important to perform an ultrasound examination to reinforce our diagnosis, which revealed a large cystic mass in the left gluteal subcutaneous plane that was excised. As a definitive management, surgery was performed with excision of the swelling, which was extracted in toto and was identified as a cyst, and on histopathological examination, the cyst wall was lined by stratified squamous epithelium. Hence, this case report highlights a rare case of a giant epidermal cyst in the gluteal region.

## Introduction

An epidermal inclusion cyst can develop over the skin of any part of the body, generally at the distal part of the hair follicle or in the sebaceous gland due to the movement of epidermal cells from the epidermis into the dermis [1], which occurs due to obstruction in the pilosebaceous glands. These types of cysts are more common on hair-bearing areas of the body like the trunk, face, scalp, and neck. Common etiology involves sun-damaged skin and acne vulgaris. Unusual sites include the breast, penis, spleen, bones, subungual region, palms, soles, and buttocks [2]. Etiology for epidermal cysts over these unusual sites is mostly due to trauma or iatrogenic injuries at specific locations like the buttocks [3]. These cysts have no genetic preponderances but can be seen in some hereditary syndromes like Gardner’s syndrome, Favre-Racouchot syndrome, and Gorlin syndrome [4,5].

Epidermal cysts are asymptomatic per se and usually present as a swelling or nodule over the skin. Patients generally visit surgeons due to cosmetic reasons; otherwise, in most patients, these swellings go unnoticed if asymptomatic [3]. Some cysts undergo acute bacterial or fungal infections, which lead to signs of inflammation and the spread of infection to surrounding areas. A progressive increase in the size of swelling with signs of inflammation at unusual sites brings the patient to the surgeon [6,7].

Here, we present a case of a young female who presented with a large swelling (consisting of a mass) in the left gluteal region. (At this point of the visual clinical examination, the nature of the mass was not determined.) The gluteal region as a site of epidermal cyst is very rarely found and is specific in patients who have a history of trauma or intramuscular injection at that site. Our patient had no significant history depicting a cause for an epidermal inclusion cyst at an unusual location, the cause of which remains idiopathic. No case report is found until now that has an idiopathic incidence of an epidermal cyst on the buttock with a large size almost covering the whole of the left buttock for only two years, thereby making our case report distinctive from other available reports.

## Case presentation

**Figure 1:**
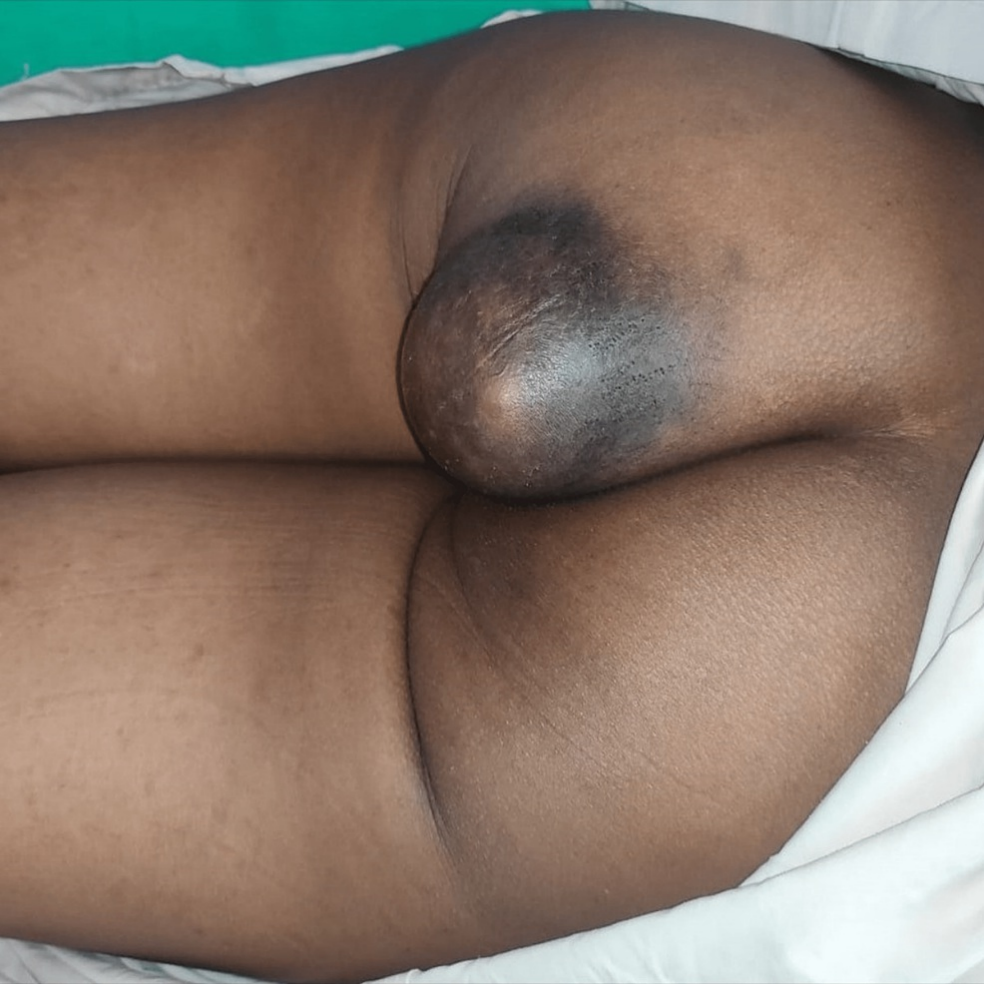
Clinical image of a giant epidermal cyst over the left gluteal region.

**Figure 2:**
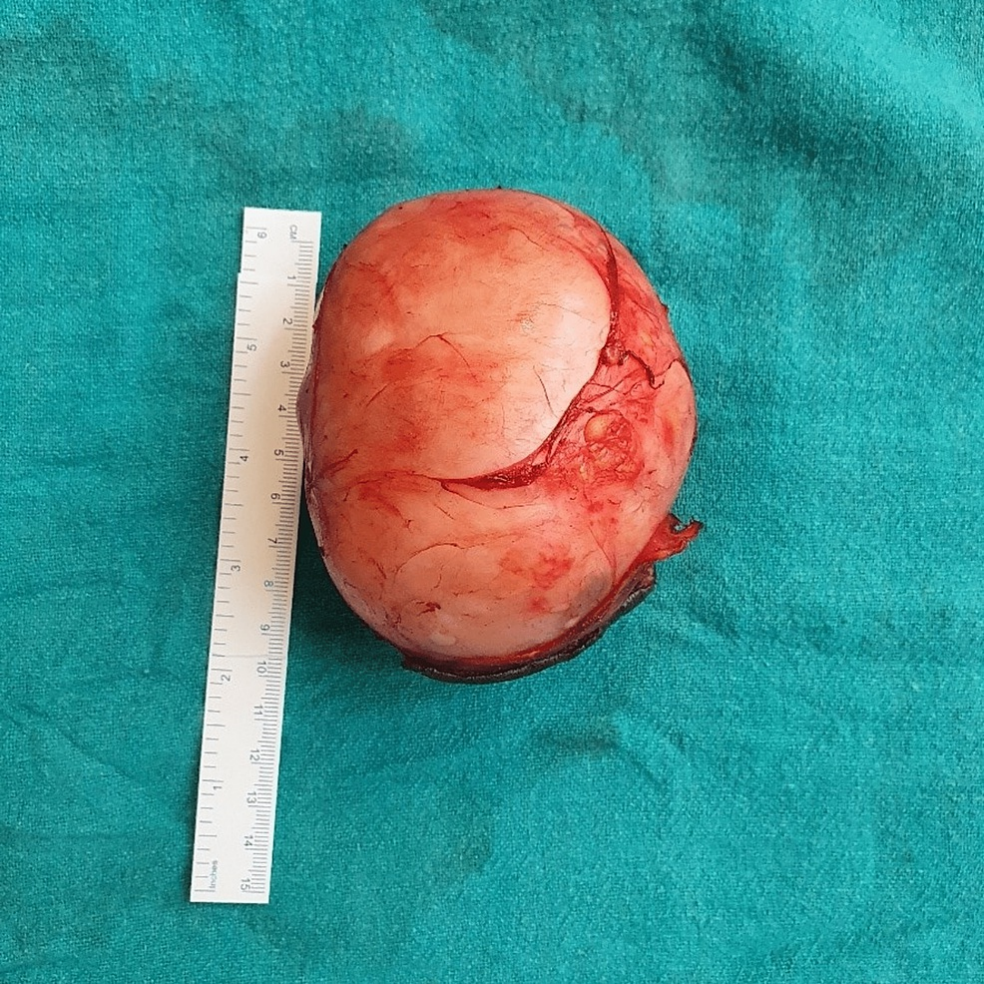
Specimen of the giant epidermal cyst excised in toto.

**Figure 3:**
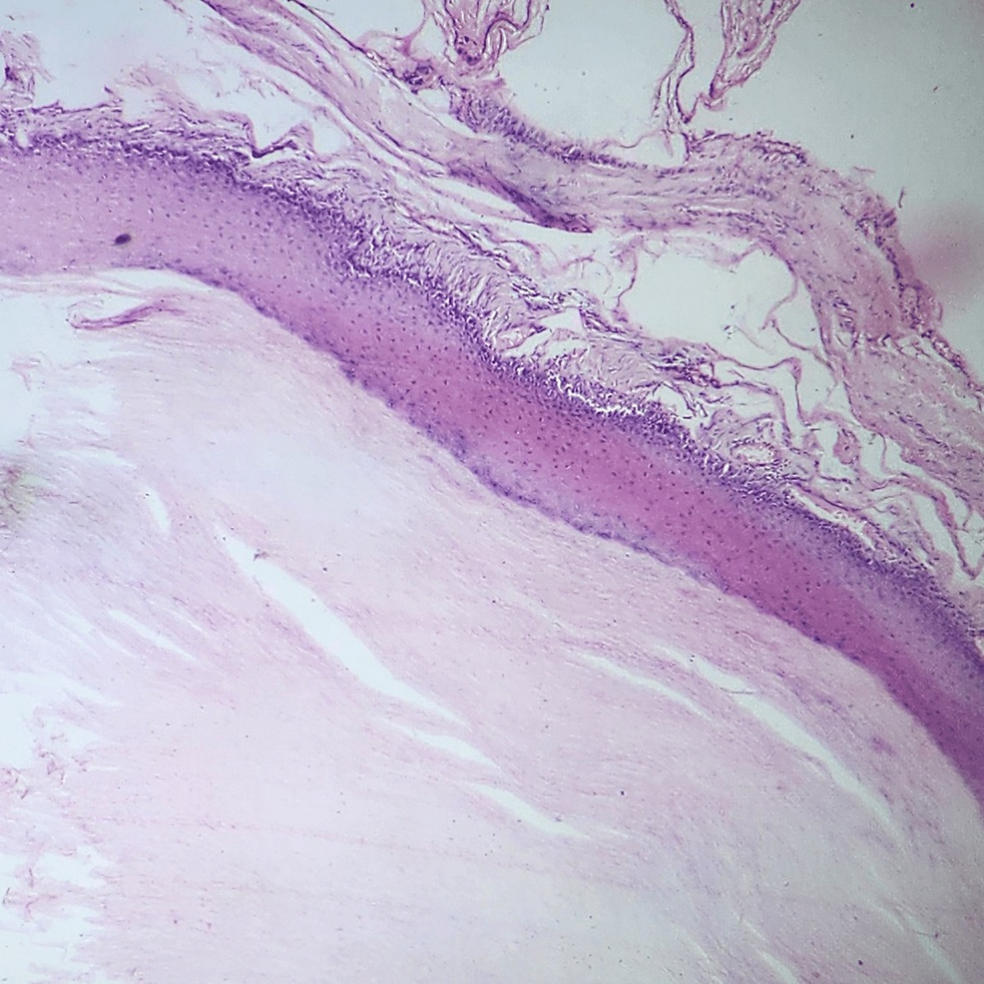
Histopathology slide showing the cyst wall lined by stratified squamous epithelium and filled with keratinaceous debris.

A 31-year-old female, a laborer by profession, reported to the outpatient clinic with complaints of swelling over the left buttock for two years, which was insidious in onset and slow-growing. The patient eventually described a discomfort that made it impossible for her to sit for long hours or sleep in the supine position. There was no significant past history related to trauma or iatrogenic injury. Clinical examination revealed a huge, soft-to-firm, regularly circumscribed mass over the left gluteal region (Figure 1), and the overlying skin looked glossy and discolored with no visible punctum on it. No local rise in temperature was noted, so an acute abscess was ruled out. There was no underlying communication with the rectum or anal canal on proctoscopic examination. Clinically, a diagnosis of giant lipoma was made, but due to its large size and involvement of the whole left buttock, it was considered important to perform an ultrasound examination to reinforce the diagnosis, which revealed a smooth-walled collection in the subcutaneous plane. As the definitive management, surgery proceeded with the excision of the swelling, which was extracted into toto and was identified as a cyst (Figure 2). An extensive elliptical skin incision over the summit of the swelling was made. Fine dissection was done to remove all fibrous attachments of the cyst to adjacent tissue. The well-encapsulated swelling was excised in toto without rupturing it. The excess skin was excised, and primary closure was achieved with mattress suturing. The excessive skin was scraped, and the edges of the residual skin were sutured together. Postoperatively, the cyst was opened, which contained an odorless, purulent, whitish-creamy material, and the cyst wall was lined by stratified squamous epithelium, which was evidently visible on histopathology (Figure 3).

## Discussion

The routine lesions found in subcutaneous tissues are kinds of cysts, of which 80% are epidermal cysts, which are found in postoperative specimens [3]. Inflammatory proliferation in sebaceous glands and hair follicles within the dermis leads to the origination of these cystic spaces occupying lesions through the epidermis of the skin [1,5]. Thus, these lesions are regularly discovered in the head, neck, and trunk, which are common sebaceous glands and hair-bearing areas of the skin. Other risk factors include human papillomavirus (HPV), congenital syndromes, and foreign body insertion into the epidermis and dermis (injections going into the wrong plane of the dermis) [1,3,5]. These cystic lesions clinically have a wide spectrum of differentials, which include lipomas, fibromas, neuromas, neurofibromatosis, etc. Clinically, these cystic lesions are firm to hard types of swelling, and they move in all planes of the dermis when palpated. Clinically smaller tumors can be easily diagnosed and labeled based on palpating and understanding the consistency of any type of skin swelling. If any dilemma develops in clinical diagnosis, we now live in an advanced era where overdiagnosis can be reinforced by radiology.

Doppler ultrasound, magnetic resonance imaging (MRI), and computed tomography are the investigations that are routinely performed to delineate these epidermal cysts. Ultrasound is easy to perform and the most cost-effective modality but is subjective in analysis. It is a preferred modality in only smaller lesions as it cannot appropriate measure the extensions of larger lesions. It also helps in the diagnosis of cysts that are present on the breast and helps in delineating the plane in which the lesion is lying [1,3,7]. Although ultrasound is very handy, the investigation of choice and gold standard for diagnosis of this lesion is MRI. It delineates the lesion and tells us about the proper plane, extension, and surrounding invasion by the lesion. It is kept reserved for large epidermal cysts and is not used routinely. Whether MRI is obligatory in any specific case is generally decided only after the clinical examination if the margins of the lesion are not palpable or have a diffuse spread or are felt to be invading surrounding structures. MRI or ultrasound can easily differentiate between the types of lesions, which are mentioned above as differential diagnoses, which sometimes are very hard to diagnose clinically. MRI depicts these cysts as well-circumscribed, oval-to-round lesions with hypointense signaling in T1-weighted images and hyperintense signaling in T2-weighted imaging [1,3,4,7]. Radiological differentials of epidermal cysts include implantation dermoid, trichilemmal cysts, cystic degeneration, vascular and neurogenic tumors, hemorrhagic lymphangioma, cystic teratoma, and ganglion cysts. MRI, by far, is the most excellent method to define neoplasms and soft-tissue tumors [3].

Medical management of this condition is not an option, and thus, the management exclusively remains to be surgical excision. Surgical elimination of these cysts has to be precise, and no spillage of contents into surrounding areas is allowed while carrying out the excision of the epidermal cyst. This prevents the recurrence and decreases the intensity of inflammation, which occurs in the postoperative phase and thus allows an enhanced recovery in the surgical site. It also safeguards us from an incidental and infrequent occurrence of malignancy that can occur in such lesions, the occurrence of which is unheard of [1,7]. An in toto abolition of the cyst wall must be carried off, and the cyst is not allowed to burst or rupture in any iatrogenic way during this process. In available documentation about epidermal cysts, even on complete dissection, a 3% recurrence rate has been reported overall. In their study on 300 patients with epidermal cysts on various sites, Bauer and Lewis concluded that there was a 2.2% recurrence, with higher chances of malignant reformation most likely into a squamous cell carcinoma [7].

Finally, histopathology remains the final gold standard investigation based on which the epidermal cyst can be dauntlessly remarked as either benign or malignant. Further, in the case of malignancy, a detailed histopathological report given margins and extensions of the cyst can flawlessly help in building up a more prompt treatment approach [8,9,10].

Epidermal cysts are common benign tumors and develop at a snail’s pace over years. All types of symptomatic cysts are managed by surgery alone. Uncomplicated cysts are easily removed by surgery, protecting surrounding anatomic tissues and organs, which are studied before surgery by imaging data available. Pericystectomy, with complete excision of the cyst wall, is the definitive treatment for epidermal cysts. In the case of rupture or secondary inflammation of these cysts, the cavity of the cysts is drained out completely with daily dressing to prompt secondary healing or sutured at a later date.

## Conclusions

Epidermal cysts are one of the most common types or variants of the cyst-like form of lesions lying in the subcutaneous plane, which are usually small-sized, slow-growing, and asymptomatic. They can appear anywhere in the body but preferably over the face, neck, and trunk, whereas unusual sites include the breast, penis, spleen, bones, subungual region, palms, soles, and buttocks. Giant epidermal inclusion over the gluteal region is extremely rare. Therefore, this case report highlighted a rare case of a giant epidermal cyst over the gluteal region, which is specific in patients who has a history of trauma or intramuscular injection at that site. But the patient, in this case, reported no significant history depicting a cause for an epidermal inclusion cyst at an unusual location, the cause of which remains idiopathic. Additionally, it is important to differentiate such benign lesions from malignancies on clinical examination and confirm using the radiological modalities available. Complete excision of the cyst is the treatment of choice that demonstrated excellent outcomes.
